# Fabrication of Polydopamine/hemin/TiO_2_ Composites with Enhanced Visible Light Absorption for Efficient Photocatalytic Degradation of Methylene Blue

**DOI:** 10.3390/polym17030311

**Published:** 2025-01-24

**Authors:** Zhuandong Zhu, Shengrong Zhou, Debin Tian, Guang-Zhao Li, Gang Chen, Dong Fang, Jiaxuan Cao, Fumei Wang, Wenyan Wang, Xuewei He, Wei Zhang

**Affiliations:** 1 Key Laboratory of Materials and Surface Technology (Ministry of Education), School of Materials Science and Engineering, Xihua University, Chengdu 610039, China; zzd1913623@163.com (Z.Z.); shengrongzhou0611@163.com (S.Z.); t793928034@163.com (D.T.); gangchen@xhu.edu.cn (G.C.); fangdongzj1101@163.com (D.F.); jiaxuancao_study@163.com (J.C.); fumei_wang0919@163.com (F.W.); wwyandmmy@163.com (W.W.); hxw124589@163.com (X.H.); 2 Engineering Research Center of Intelligent Air-Ground Integration Vehicle and Control (Xihua University), Ministry of Education, Chengdu 610039, China; 3 State Key Laboratory of Polymer Materials Engineering, Polymer Research Institute, Sichuan University, Chengdu 610065, China

**Keywords:** TiO_2_, photocatalytic degradation, hemin, PDA, methylene blue

## Abstract

With the rapid progression of industrialization, water pollution has emerged as an increasingly critical issue, especially due to the release of organic dyes such as methylene blue (MB), which poses serious threats to both the environment and human health. Developing efficient photocatalysts to effectively degrade these pollutants is therefore of paramount importance. In this work, titanium dioxide (TiO_2_) was modified with the photosensitizer hemin and the hydroxyl-rich polymer polydopamine (PDA) to enhance its photocatalytic degradation performance. Hemin and PDA function as photosensitizers, extending the light absorption of TiO_2_ into the visible spectrum, reducing its bandgap energy, and effectively promoting separation of photogenerated electron–hole pairs through conjugated structures. Additionally, the strong adhesion of PDA enabled the rapid transfer and effective utilization of photogenerated electrons, while its abundant phenolic hydroxyls increased MB adsorption on the photocatalyst’s surface. Experimental results demonstrated a significant enhancement in photocatalytic activity, with the 1%PDA/3%hemin/TiO_2_ composite achieving degradation rates of 91.79% under UV light and 71.53% under visible light within 120 min, representing 2.22- and 2.05-fold increases compared to unmodified TiO_2_, respectively. This research presents an effective modification approach and provides important guidance for designing high-performance TiO_2_-based photocatalysts aimed at environmental remediation.

## 1. Introduction

The swift growth of industrial activities has heavily polluted natural water sources with organic dyes and heavy metals, creating serious risks to both human health and ecosystems [1,2,3,4,5]. To address this pressing issue, various remediation methods, including biodegradation and photocatalytic degradation, have been extensively studied [6,7,8,9]. Among these, photocatalytic degradation is considered the most effective due to its rapid oxidation process, ease of reuse, and simple post-treatment requirements [10,11,12,13]. Titanium dioxide (TiO_2_) has emerged as a widely used photocatalyst because of its environmental friendliness, cost-effectiveness, and easy availability [14,15,16]. Beyond photocatalytic degradation, TiO_2_ also exhibits promising potential in hydrogen generation, self-cleaning, and sterilization applications [17,18]. Among its three crystalline forms including anatase, rutile, and brookite, anatase TiO_2_ has attracted particular attention due to its high stability, facile synthesis, and excellent photocatalytic activity [19,20,21]. The photocatalytic process of TiO_2_ begins with light energy surpassing its bandgap, enabling electron transfer from the valence band to the conduction band. This process leaves behind positively charged holes in the valence band, which can react with water and oxygen to produce highly reactive oxygen species (ROS) that degrade organic pollutants. However, anatase TiO_2_ has a wide bandgap (approximately 3.2 eV) and is responsive only to ultraviolet (UV) light with a wavelength of 387.5 nm. Since UV light accounts for merely 5% of the solar spectrum, the practical application of TiO_2_ under natural sunlight is significantly restricted [22,23,24]. To overcome this limitation, numerous studies have focused on modifying TiO_2_, aiming to expand its absorption range to the visible light region by adjusting its band structure, introducing defects or doping heterogeneous elements, thereby improving the utilization of sunlight and enhancing its photocatalytic performance [25,26,27,28]. One promising approach is the use of organic photosensitizers, which can sensitize TiO_2_, making visible light activation possible and aiding in the decomposition of various organic contaminants [29,30,31,32,33].

Apart from expanding the light absorption range, another effective strategy to improve the photocatalytic activity of TiO_2_ is to ensure the rapid transfer and efficient consumption of photogenerated electrons. To maximize efficiency, electrons must quickly migrate to the photocatalyst surface for reactions with pollutants, and the photocatalyst should strongly adsorb the target contaminants [34,35]. This strategy minimizes electron–hole recombination while boosting energy utilization efficiency, ultimately improving the overall photocatalytic performance. The combination of TiO_2_ with polymers has emerged as a promising method to achieve these goals. Unlike inorganic compounds, polymers can form covalent bonds, ionic bonds, and hydrogen bonds with photocatalysts, resulting in stronger interfacial interactions [13,36,37]. These interactions facilitate rapid electron transfer across the interface, enabling photogenerated electrons to reach the surface more efficiently, where they can react with adsorbed pollutants.

Enhancing the photocatalytic performance of TiO_2_ can be efficiently achieved by modifying or doping it with a variety of organic or inorganic compounds. For example, Milad Neshastehgar synthesized TiO_2_@Silane@SiO_2_ photocatalyst materials, which exhibited improved absorption in the visible light region and efficient photocatalytic degradation of methylene blue (MB) [38]. Similarly, Ricardo M. S. Sendão et al. developed carbon dots-TiO_2_ composites, achieving a remarkable 367% increase in catalytic performance [39]. Substantial advancements have been achieved in enhancing the photocatalytic efficiency of TiO_2_ via diverse modifications, particularly in enhancing its visible light absorption and catalytic efficiency. However, further advancements are needed to optimize charge separation, strengthen interfacial interactions, and improve the adsorption of target pollutants, especially under practical operating conditions.

In this study, titanium dioxide (TiO_2_) was modified by incorporating a photosensitizer, hemin, and a hydroxyl-rich polymer, namely polydopamine (PDA), to enhance its photocatalytic degradation performance, particularly in the visible light region. Hemin can be used as a photosensitizer and electron mediator, capturing visible light to enhance energy transfer and facilitate the charge separation, which drives photocatalytic reactions. Meanwhile, PDA broadens TiO_2_’s light absorption range and facilitates faster electron transfer to the surface, optimizing hole utilization. Additionally, the abundant phenolic hydroxyl groups in PDA enhance the adsorption of methylene blue (MB), providing more reactive sites for photocatalytic degradation. By adjusting the mass fractions of PDA and hemin, we optimized the performance of the photocatalyst, resulting in a significantly enhanced degradation rate of MB under both ultraviolet and visible light for 1%PDA/3%hemin/TiO_2_. Additionally, recycling experiments demonstrated the outstanding stability and reusability of the synthesized composites, highlighting their potential for practical wastewater treatment applications. This study highlights the potential of combining photosensitizers and polymers to achieve synergistic enhancements in photocatalytic performance, providing valuable insights for the development of high-performance TiO_2_-based photocatalysts.

## 2. Materials and Methods

### 2.1. Materials

**Scheme 1:**
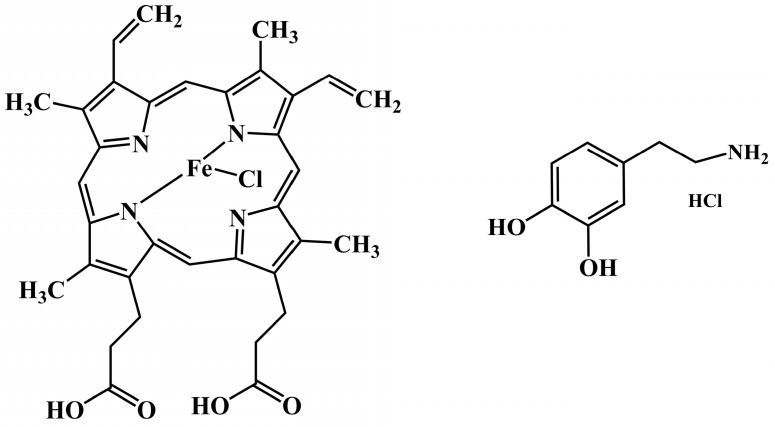
The chemical structures of hemin and dopamine hydrochloride.

Hemin was purchased from Jiuding Chemical (Shanghai) Science and Technology Co., Ltd. (Shanghai, China). Other chemicals, including butyl titanate, methylene blue, glacial acetic acid, sodium hydroxide, and deionized water, were obtained from Chengdu Kelong Chemical Reagents Co., Ltd. (Chengdu, China). Absolute ethanol and dopamine hydrochloride were supplied by Shanghai Taitan Science and Technology Co., Ltd. (Shanghai, China). All reagents were of analytical grade and used directly without further purification. The chemical structures of hemin and dopamine hydrochloride are shown in Scheme 1.

### 2.2. Preparation of Neat hemin/TiO_2_ Particles

To prepare TiO_2_ particles, 20 mL of butyl titanate was added to 70 mL of absolute ethanol in a beaker. In a separate beaker, 8 mL of glacial acetic acid was mixed with a solution of 70 mL of absolute ethanol and 20 mL of deionized water. The exception was that 3 wt% of hemin was dissolved in a mixture of absolute ethanol and deionized water before being incorporated into the process. The glacial acetic acid solution was then gradually added to the butyl titanate solution under continuous stirring. The resulting mixture was stirred mechanically for 20 min and subsequently heated at 50 °C for another 20 min. A gray gel was formed, which was collected and dried in an oven at 100 °C. Finally, the dried gel was calcined at 260 °C for 2 h to obtain TiO_2_ powder.

### 2.3. Preparation of PDA/hemin/TiO_2_ Composites

To prepare PDA/TiO_2_, 1 g of TiO_2_ particles was dispersed in 50 mL of deionized water using ultrasonic treatment for 30 min. The pH of the suspension was adjusted to 8.5 by adding a sodium hydroxide solution. Dopamine hydrochloride was then introduced into the mixture in appropriate amounts, followed by vigorous stirring. The resulting precipitates were collected by filtration and dried in a blast oven at 80 °C for 12 h to obtain the powders. These powders were labeled as 0.1%PDA/TiO_2_, 0.5%PDA/TiO_2_, 1%PDA/TiO_2_, 2%PDA/TiO_2_, and 3%PDA/TiO_2_, depending on the amount of dopamine hydrochloride used. Note that, in our previous study, 3%hemin concentration achieved the best balance between enhanced adsorption and avoiding agglomeration. Excess hemin causes particle agglomeration, reducing surface area and performance. Therefore, 3%hemin was selected as the optimal concentration for modification in this study [40]. PDA/3%hemin/TiO_2_ composite powder was prepared using the same method, with the addition of 1% PDA and 3%hemin/TiO_2_. The as-prepared composite powders were named as 0.1% PDA/3%hemin/TiO_2_, 0.5% PDA/3%hemin/TiO_2_, 1%PDA/3%hemin/TiO_2_, 2%PDA/3%hemin/TiO_2_, and 3%PDA/3%hemin/TiO_2_.

### 2.4. Characterization

The crystalline properties of the synthesized samples were analyzed using a high-performance X-ray diffractometer (XRD, DX-2700, Dan Dong Haoyuan Instrument Co., Ltd., Dandong, China) with Cu Kα radiation. The XRD measurements were performed at 45 kV, scanning at a speed of 0.01°/s across a range of 10° to 80°. Raman spectra were obtained using an iHR320 spectrometer (HORIBA) with a 532 nm Argon-ion laser as the excitation source. The surface morphology of the powders was examined via scanning electron microscopy (SEM, Hitachi SU8010, Tokyo, Japan) at 10 kV under vacuum conditions, after sputtering the samples with a thin layer of gold. UV–Vis diffuse reflectance spectra (DRS) were recorded on a Lambda 1050 spectrophotometer (PerkinElmer, Waltham, MA, USA) across the wavelength range of 200–800 nm, using BaSO_4_ as the reference material.

### 2.5. Photocatalytic Performance Tests

The photocatalytic degradation efficiency of the prepared samples was evaluated using methylene blue (MB) as a model pollutant. In each test, 60 mL of MB solution and 200 mg of photocatalyst were added to a 100 mL beaker. The mixture was stirred in the dark to establish adsorption equilibrium before exposure to light. During irradiation, 3–4 mL of the suspension was withdrawn at regular intervals and centrifuged, and 1 mL of the supernatant was analyzed to determine the MB concentration. The degradation efficiency under both UV and visible light was tested using the same procedure.

UV irradiation experiments were conducted using a Jiancai ZW30S19W (Shenzhen Anhongda Optoelectronics Technology Co., Ltd., Foshan, China) germicidal lamp (33 W) emitting at a wavelength of 253.7 nm. The lamp, with dimensions of 894.6 mm in length and 19 mm in diameter, was positioned 10 cm above the sample to ensure uniform illumination. For visible light irradiation, a Philips R7s (Philips Investment Co., Ltd., Shanghai, China) tungsten iodide lamp (500 W) was employed, providing a peak wavelength near 799.5 nm, with the same 10 cm distance maintained to the sample. The concentration of methylene blue (MB) was monitored at 664 nm using UV-Vis spectrophotometry, and its adsorption and degradation efficiencies were calculated based on the Lambert–Beer law, as shown in Equation (1) [19,41].

(1) $$A=\mathrm{lg}\left(\frac{I_{0}}{I}\right)=lg\left(\frac{1}{T}\right)=\varepsilon bc$$

where *A* is the absorbance, *I*_0_ is the initial light intensity, *I* is the transmitted light intensity, *T* is the transmittance, *ε* is the molar absorption coefficient, *b* is the solution thickness, and *c* is the MB concentration. Under fixed conditions, the solution concentration *c* is directly proportional to its absorbance *A*. The degradation efficiency *η* was calculated using Equation (2):

(2) $$\eta =\frac{c_{0}-c}{c_{0}}\times 100\%=\frac{A_{0}-A}{A_{0}}100\%$$

where *c*_0_ and *c* represent the initial and remaining MB concentration, respectively, and *A*_0_ and *A* denote the initial and resultant absorbance values, respectively.

## 3. Results

### 3.1. Crystallization Behavior of PDA/3%hemin/TiO_2_

**Figure 1:**
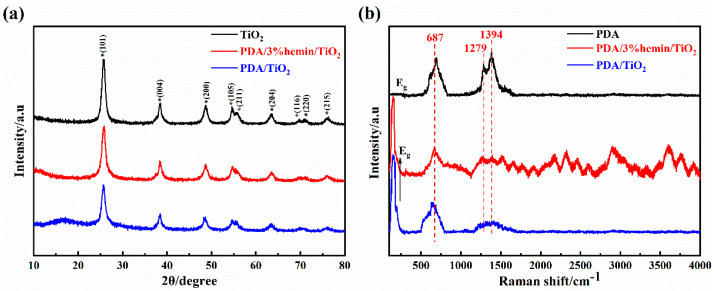
(**a**) XRD patterns of TiO_2_, PDA/TiO_2_ and PDA/3%hemin/TiO_2_ powder samples. (**b**) Raman spectra of PDA, PDA/TiO_2_ and PDA/3%hemin/TiO_2_ powder samples.

The anatase phase of TiO_2_ is essential for high photocatalytic efficiency due to its unique electronic structure, which enables efficient charge separation and significantly enhances the generation of reactive oxygen species [42,43,44]. The XRD patterns of TiO_2_, PDA/TiO_2_, and PDA/3%hemin/TiO_2_ composite powder are shown in Figure 1a. As can be seen, all samples exhibit characteristic diffraction peaks of anatase TiO_2_ located at 2θ ≈ 25.3°, corresponding to (101) plane, confirming the retention of the anatase phase after modification. The peak intensity of PDA/TiO_2_ and PDA/3%hemin/TiO_2_ is slightly reduced compared to pure TiO_2_, probably attributed to the fact the PDA cover the surface of TiO_2_, thereby reducing the penetration of X-ray. Furthermore, results of the Raman spectroscopy of the samples are presented in Figure 1b. As can be seen, the Raman spectra of PDA/TiO_2_ and PDA/hemin/TiO_2_ closely resemble those of PDA within the range of 500–2000 cm^−1^. Two characteristic bands of anatase TiO_2_ are observed at 154 cm^−1^ (Eg) and 196 cm^−1^ (Eg), while other low-intensity bands are overlapped by the strong signal bands from PDA and hemin [21,45]. Notably, the bands at 687, 1279, and 1394 cm^−1^ observed in all samples are attributed to the C-H bonds, benzoquinone, and C=C bonds within the benzene rings of PDA, respectively. Additionally, the broad multiple bands in the 1500–4000 cm^−1^ range are associated with the absorption of hemin. These results demonstrate the successful incorporation of PDA and hemin into TiO_2_ without disrupting the anatase crystalline structure, which is consistent with the XRD results.

### 3.2. Morphologies of PDA/3%hemin/TiO_2_

**Figure 2:**
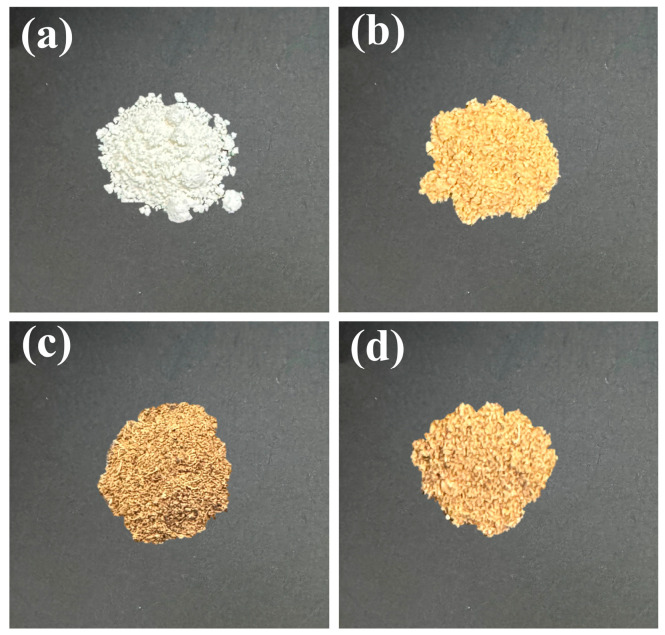
Digital photos of TiO_2_ (**a**), PDA/TiO_2_ (**b**), 3%hemin/TiO_2_ (**c**) and PDA/3%hemin/TiO_2_ (**d**).

**Scheme 2:**
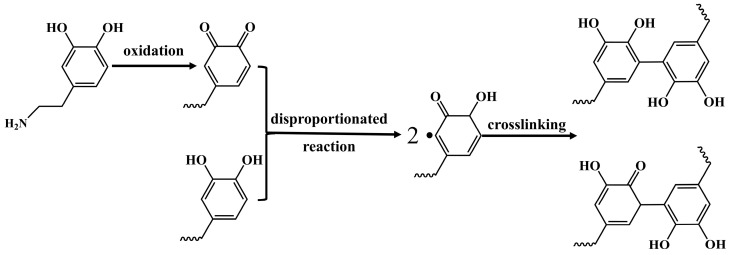
The crosslinking reaction of PDA.

Figure 2 presents the digital images of TiO_2_ and its modified samples, illustrating the visible color change caused by PDA and hemin modifications. With the incorporation of PDA, PDA/TiO_2_ shows a yellow color which can be attributed to the benzoquinone and biphenyl conjugated structures in PDA, as shown in Scheme 2. The 3%hemin/TiO_2_ sample exhibits a dark gray color, due to the conjugated structure of hemin with a central Fe^3+^ ion, as shown in Scheme 1, significantly enhancing visible light absorption. Finally, the PDA/3%hemin/TiO_2_ composite powder shows a dark brown color due to the synergistic effect of PDA and hemin, which together further improve the visible light absorption capacity.

**Figure 3:**
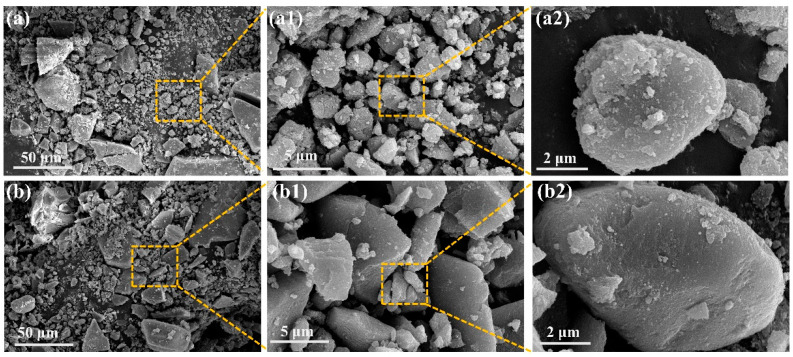
SEM images of PDA/3%hemin/TiO_2_ composite powder (**a**,**a1**,**a2**) and PDA/TiO_2_ (**b**,**b1**,**b2**).

Figure 3 shows the SEM images of surface morphologies of PDA/TiO_2_ and PDA/3%hemin/TiO_2_. In both samples, the particles exhibit an irregular shape with sizes in the micrometer range, as shown in Figure 3a,b. In addition, the surface of PDA/TiO_2_ appears relatively smooth, reflecting the uniform coating of PDA. In contrast, the surface of PDA/3%hemin/TiO_2_ exhibits noticeably increased roughness. The enhanced surface roughness could provide additional active sites for photocatalytic reactions, improving overall photocatalytic efficiency.

**Figure 4:**
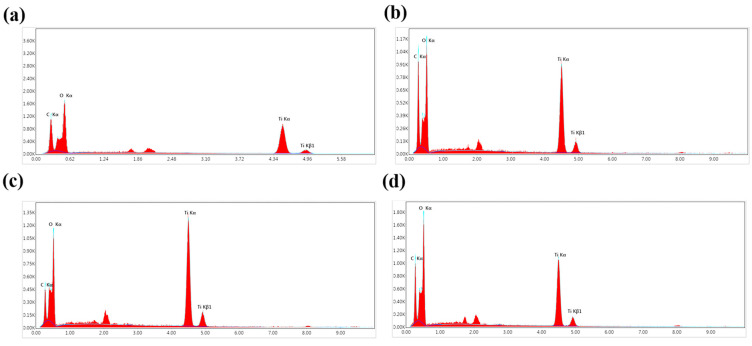
EDS spectra of TiO_2_ (**a**), PDA/TiO_2_ (**b**), 3%hemin/TiO_2_ (**c**), and PDA/3%hemin/TiO_2_ (**d**) PDA/3%hemin/TiO_2_. The blue color represents the fitting line, the red color represents the peak area, and the navy blue color curve at the bottom of the peak represents the background.

Figure 4 shows the EDS spectra of various composite powders, and their corresponding mass fraction of the element is presented in Table 1. In pure TiO_2_, the C content is 21.5%, while in PDA/TiO_2_, the C content decreases to 18.45% and the O content increases significantly to 45.09%, indicating the formation of an oxygen-rich shell layer by PDA on the TiO_2_ surface. For 3%hemin/TiO_2_, the C content further decreases to 8.12% and the Ti content increases to 47.52%, suggesting that hemin modification exposes more TiO_2_ surfaces. In PDA/3%hemin/TiO_2_, the C content rises to 12.25% and the O content reaches 49.79%, indicating an additional PDA coating on the surface of 3%hemin/TiO_2_, forming a multilayer structure with TiO_2_ as the core and PDA and hemin as the shell. This structure not only increases the proportion of oxygen-containing groups on the surface but also enhances interfacial interactions, benefiting to the photocatalytic degradation properties.

### 3.3. Ultraviolet–Visible Scattering and Bandgap Energy

**Figure 5:**
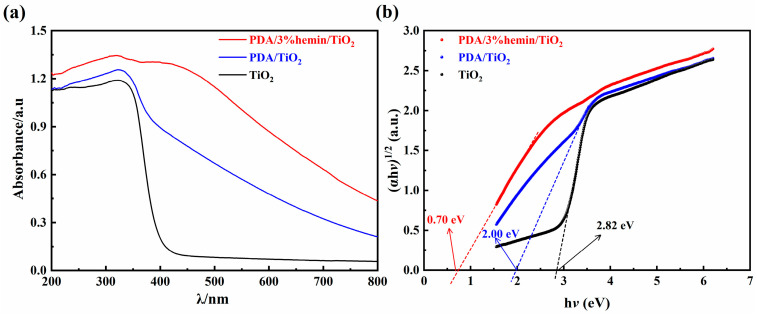
(**a**) UV-Vis DRS of TiO_2_, PDA/TiO_2_, and PDA/3%hemin/TiO_2_ and (**b**) the corresponding Tauc plots.

As shown in Figure 5a, pristine TiO_2_ exhibits strong absorption only in the ultraviolet region, with a sharp cutoff at wavelengths above 400 nm, indicating its inherent limitation of absorbing solely UV light. This behavior is consistent with its wide bandgap energy and restricts its practical application under visible light. Upon modification with PDA, the light absorption of PDA/TiO_2_ significantly increases across the entire measured spectrum, particularly in the visible light range. This enhancement is attributed to the presence of PDA, which contains conjugated benzoquinone and biphenyl structures capable of extending the absorption edge into the visible region, as reflected in its darker color. Further incorporation of hemin into PDA/TiO_2_ leads to a substantial improvement in visible light absorption. This enhancement is due to the conjugated structure of hemin and its central Fe^3+^, which collectively act as a photosensitizer to broaden the spectral response. The synergistic effect of PDA and heme endows PDA/3% heme/TiO_2_ composite with the highest absorbance among all the samples, which is beneficial to the generation of photogenerated electron–hole pairs under visible light irradiation. Figure 5b shows the Tauc plots derived from diffuse reflectance spectra (DRS) data, which were further used to calculate the bandgap energy of the samples based on the Kubelka–Munk equation.

(3) $${\left(\alpha hv\right)}^{2}=A\;{\left(hv-E_{g}\right)}^{n}$$

where α represents the absorption coefficient, h is the Planck’s constant, v is the optical frequency, *A* is the constant, and *E_g_* is the bandgap energy. For TiO_2_, n = 4, which is consistent with its indirect bandgap nature [33,46,47]. For pristine TiO_2_, the bandgap energy was determined to be 2.82 eV, consistent with its ultraviolet light absorption. In contrast, the incorporation of PDA and hemin significantly reduced the bandgap energy to 0.90 eV for PDA/TiO_2_ and 0.70 eV for PDA/3%hemin/TiO_2_, respectively. This remarkable reduction in Eg indicates that the modified photocatalysts can absorb light with lower energy, thereby extending their usability into the visible light range. Such a remarkable decrease in bandgap energy can be attributed to the large conjugated structures in both PDA and hemin. These structures effectively act as photosensitizers, absorbing lower-energy light and transferring the excitation energy to the TiO_2_ matrix. This process enhances the separation and mobility of photogenerated electron–hole pairs on the TiO_2_ surface, which is crucial for improving the photocatalytic efficiency.

### 3.4. The Adsorption of Pollutants and Photocatalytic Degradation Properties

**Scheme 3:**
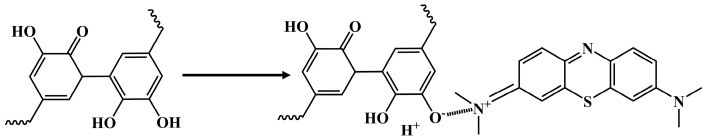
The proposed interaction between PDA and MB.

**Figure 6:**
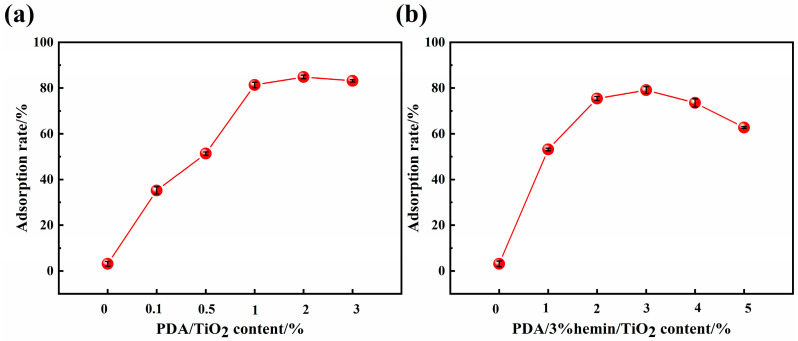
The adsorption rate of PDA/TiO_2_ (**a**) and PDA/hemin/TiO_2_ (**b**).

The adsorption properties of PDA/TiO_2_ and PDA/3%hemin/TiO_2_ composite powder were evaluated, and the results are presented in Figure 6. The introduction of PDA significantly enhances the adsorption affinity for MB, with the adsorption rate reaching a maximum of 84.85% at 2 wt% PDA/TiO_2_, as presented in Figure 6a. This value is notably higher than those reported for most TiO_2_ modified with inorganic compounds, highlighting the superior adsorption capability endowed by PDA. The enhanced adsorption is attributed to the ionic interactions between the phenolic hydroxyl groups of PDA and the cationic nitrogen species in MB, as illustrated in Scheme 3, facilitating the effective adsorption of MB onto the PDA-modified TiO_2_ surface. For the PDA/3% heme/TiO_2_ composite powders, as shown in Figure 6b, the adsorption behavior shows a slightly different trend. The sample reaches the highest adsorption rate of 79.1% at 1% PDA. However, further increase in PDA instead results in a slight decrease in adsorption. This decrease may be due to the reduced stability of the PDA/3% heme/TiO_2_ particles in solution, where excess PDA promotes particle aggregation and precipitation. In addition, as observed in the SEM images, the relatively smooth surface of PDA/3% heme/TiO_2_ may reduce the active sites available for MB adsorption, thereby limiting the overall efficiency.

**Figure 7:**
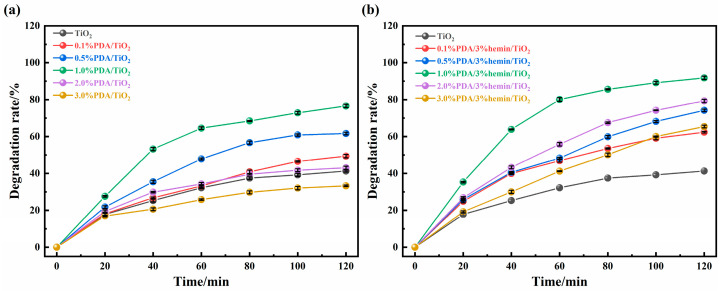
Photocatalytic degradation properties of TiO_2_, PDA/TiO_2_ (**a**) and PDA/3%hemin/TiO_2_ (**b**) under UV irradiation.

Figure 7 illustrates the photocatalytic degradation properties of TiO2, PDA/TiO_2_ and PDA/3%hemin/TiO_2_ under UV irradiation. As shown in Figure 7a, the photocatalytic degradation rate of PDA/TiO_2_ initially increases with the amount of PDA, reaching a maximum value of 76.54% after 120 min for 1%PDA/TiO_2_. This enhancement can be attributed to three synergistic factors: (1) PDA functions as a photosensitizer, effectively narrowing the bandgap energy of the composites and enhancing their UV light absorption; (2) the abundant hydroxyl groups in PDA exhibit strong affinity for MB, promoting its adsorption onto the composite surface; and (3) PDA ensures excellent interfacial adhesion, facilitating the efficient transfer of photogenerated electrons to the catalyst surface, thereby extending the catalyst’s service life and enabling it to maintain high MB degradation activity over multiple cycles [48,49,50,51]. However, excessive PDA loading leads to significant particle aggregation, reducing the available surface area, and limits contact between MB molecules and the catalyst, resulting in a notable decrease in photocatalytic activity. Specifically, when the PDA content was increased to 3%, the rates decreased significantly to 33.27% and 65.39%, respectively, which can well be attributed to the agglomeration of PDA. A similar trend was observed in the PDA/3%hemin/TiO_2_ system, as shown in Figure 7b. The photocatalytic degradation rate peaks at 91.79% for 1%PDA/3%hemin/TiO_2_ after 120 min, which is 2.22 times higher than that of pristine TiO_2_. Such superior performance is attributed to the combined effects of PDA and hemin. Hemin acts as an additional photosensitizer, introducing a large conjugated structure that enhances visible light absorption and further facilitates electron transfer. Additionally, hemin provides active sites that complement the photocatalytic processes driven by PDA. However, similar to PDA/TiO_2_, excessive PDA and hemin loading beyond the optimal 1% results in aggregation and reduced photocatalytic efficiency.

**Figure 8:**
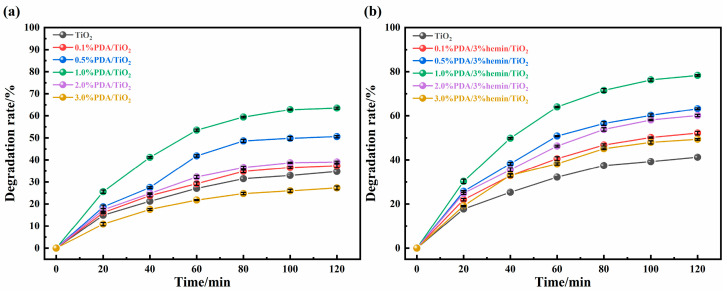
Photocatalytic degradation properties of TiO_2_, PDA/TiO_2_ (**a**) and PDA/3%hemin/TiO_2_ (**b**) under visible light.

The photocatalytic degradation performance of TiO_2_, PDA/TiO_2_, and PDA/3%hemin/TiO_2_ under visible light irradiation is shown in Figure 8. As depicted in Figure 8a, 1%PDA/TiO_2_ achieves the highest photocatalytic degradation rate of 63.55% after 120 min, which is 1.83 times higher than that of pristine TiO_2_. Similarly, as shown in Figure 8b, 1%PDA/3%hemin/TiO_2_ exhibits the highest degradation rate of 71.53%, representing a 2.05-fold improvement compared to neat TiO_2_. These results highlight the significant role of PDA and hemin in enhancing the visible-light-driven photocatalytic performance of TiO_2_.

**Figure 9:**
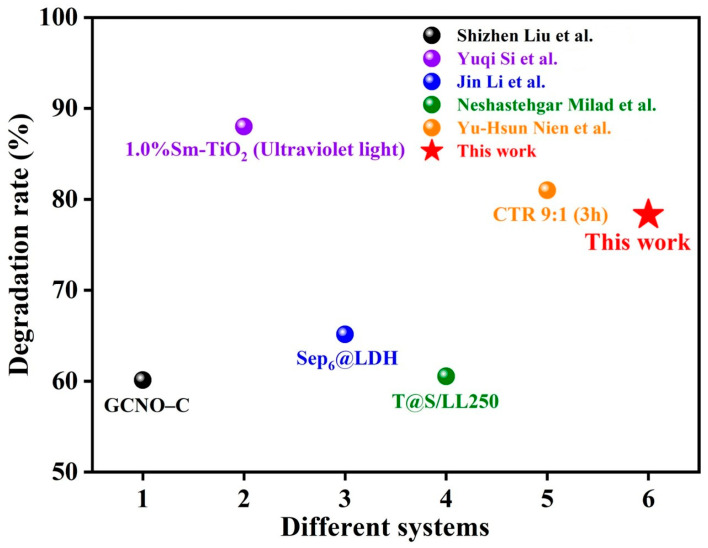
Comparison of the photocatalytic degradation efficiency of PDA/3%hemin/TiO_2_ composite powder with recently reported TiO_2_-based photocatalysts for MB degradation under visible light [38,52,53,54,55].

As shown in Figure 9, the PDA/3%hemin/TiO_2_ composite powder demonstrates excellent MB degradation efficiency, achieving a degradation rate comparable to or exceeding that of several TiO_2_-based photocatalysts reported in recent studies [38,52,53,54,55]. This highlights the significant potential of our composite in advancing photocatalytic performance under visible light conditions.

**Figure 10:**
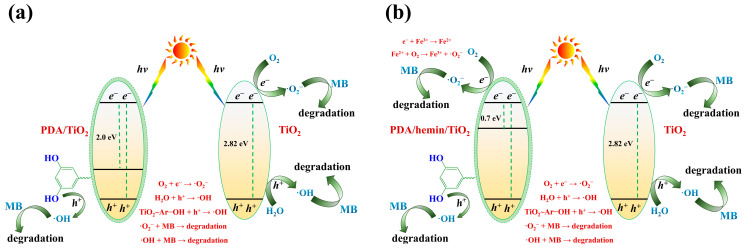
Proposed degradation mechanism of MB by PDA/TiO_2_ (**a**) and PDA/3%hemin/TiO_2_ (**b**).

The enhancement mechanisms are proposed in Figure 10. The introduction of PDA and hemin brings phenolic hydroxyl groups and Fe^3+^ into the system, which act as active sites during photocatalysis. When visible light energy is absorbed, the catalyst surface generates photogenerated electron–hole pairs, initiating photocatalytic activity. The holes oxidize the phenolic hydroxyl groups into phenoxy radicals, which further react with hydroxyl ions in water to produce highly reactive hydroxyl radicals, initiating the rapid degradation of MB [56,57]. On the other hand, the photogenerated electrons reduce Fe^3+^ to Fe^2+^, which facilitates the reduction of dissolved oxygen to form superoxide radicals. These superoxide anion radicals also contribute to the effective degradation of MB. When comparing the degradation rates of the composite photocatalysts under UV and visible light irradiation, it is evident that the photocatalytic degradation rates under visible light are significantly lower than those under UV light. This discrepancy is primarily due to the higher intensity and energy of UV light, which can be more effectively utilized by photocatalysts compared to visible light. Additionally, the reduced degradation efficiency under visible light may also be attributed to the limited portion of visible light that can be absorbed and converted by the composite photocatalyst, despite the modifications introduced by PDA and hemin.

**Figure 11:**
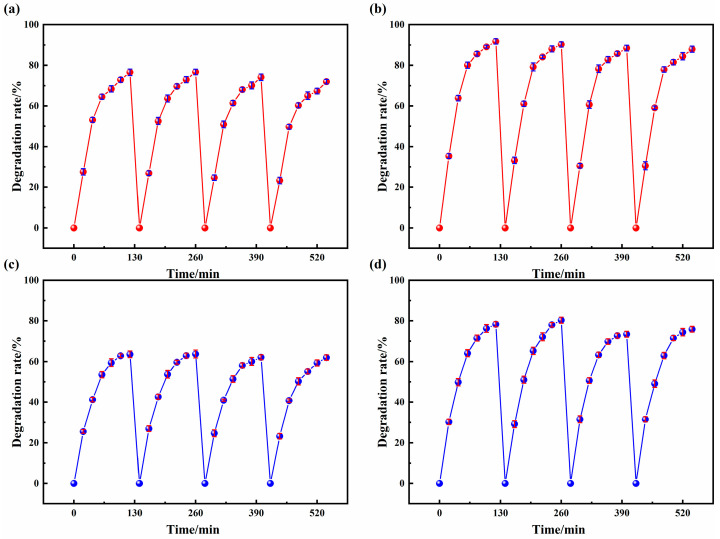
Photocatalytic cycle experiments of PDA/TiO_2_ under ultraviolet light (**a**) and visible light (**c**), and PDA/3%hemin/TiO_2_ under ultraviolet light (**b**) and visible light (**d**).

Figure 11 presents the photocatalytic cycle experiments of PDA/TiO_2_ and PDA/3%hemin/TiO_2_ under both UV and visible light. The results highlight the excellent reusability and stability of the composite photocatalysts over multiple cycles of MB degradation. As can be seen, both PDA/TiO_2_ and PDA/3%hemin/TiO_2_ exhibit consistent photocatalytic performance across four cycles under both UV and visible light irradiation. Notably, no significant decrease in degradation efficiency is observed, indicating that the structural integrity and catalytic functionality of the composites remain intact after repeated use. This stability can be attributed to the strong interfacial adhesion between PDA, hemin, and TiO_2_, which minimizes catalyst leaching and aggregation during the reaction. The preservation of photocatalytic performance across cycles is a crucial characteristic for practical applications, as it ensures that the catalysts can be reused multiple times without significant loss of efficiency, offering both economic and environmental benefits. The stability demonstrated in these experiments further confirms the potential of PDA/TiO_2_ and PDA/3%hemin/TiO_2_ as robust photocatalysts for long-term environmental remediation, providing an effective and sustainable solution for wastewater treatment.

## 4. Conclusions

To summarize, we successfully modified TiO_2_ using the photosensitizer hemin and the hydroxyl-rich polymer PDA in this work. The modification did not alter the crystalline form of TiO_2_, ensuring the retention of its anatase phase. SEM results confirmed successful PDA coating on the TiO_2_ surface, as evidenced by the additional roughness introduced for the PDA/3%hemin/TiO_2_ sample. The incorporation of hemin and PDA expanded the light absorption range into the visible region and significantly reduced the bandgap energy, with PDA/TiO_2_ and PDA/3%hemin/TiO_2_ exhibiting bandgap energies of 0.90 eV and 0.70 eV, respectively. Moreover, the strong adhesion provided by PDA facilitated the rapid transfer of photogenerated electrons to the catalyst surface, enhancing the utilization of holes, while the phenolic hydroxyls in PDA increased the adsorption of methylene blue, further improving photocatalytic efficiency. As a result, when TiO_2_ was modified with 3%hemin and 1% PDA, the degradation rate of methylene blue under both UV and visible light doubled, reaching a degradation rate of 91.79% under UV light. This work provides a promising modification strategy and valuable insights for the development of high-performance TiO_2_-based photocatalysts for environmental applications.

## Figures and Tables

**Table 1:** The mass fraction of the element in the sample.

Element	TiO_2_	PDA/TiO_2_	3%Hemin/TiO_2_	PDA/3%Hemin/TiO_2_
C	21.5	18.45	8.12	12.25
O	36.44	45.09	44.35	49.79
Ti	42.06	36.46	47.52	37.96

## Data Availability

The original contributions presented in this study are included in the article. Further inquiries can be directed to the corresponding authors.
